# Psilocybin-Induced Mystical-Type Experiences are Related to Persisting Positive Effects: A Quantitative and Qualitative Report

**DOI:** 10.3389/fphar.2022.841648

**Published:** 2022-03-09

**Authors:** Drummond E-Wen McCulloch, Maria Zofia Grzywacz, Martin Korsbak Madsen, Peter Steen Jensen, Brice Ozenne, Sophia Armand, Gitte Moos Knudsen, Patrick MacDonald Fisher, Dea Siggaard Stenbæk

**Affiliations:** ^1^ Neurobiology Research Unit and NeuroPharm, Copenhagen University Hospital Rigshospitalet, Copenhagen, Denmark; ^2^ Department of Public Health, Section of Biostatistics, University of Copenhagen, Copenhagen, Denmark; ^3^ Institute of Clinical Medicine, University of Copenhagen, Copenhagen, Denmark; ^4^ Department of Psychology, University of Copenhagen, Copenhagen, Denmark

**Keywords:** psychedelic, psilocybin, qualitative, psychology, natural language processing, mystical, phenomenology, persisting effects

## Abstract

Psychedelic drugs such as psilocybin have shown substantial promise for the treatment of several psychiatric conditions including mood and addictive disorders. They also have the remarkable property of producing persisting positive psychological changes in healthy volunteers for at least several months. In this study (NCT03289949), 35 medium-high doses of psilocybin were administered to 28 healthy volunteers (12 females). By the end of the dosing day, participants reported the intensity of their acute experience using the 30-item Mystical Experience Questionnaire (MEQ) and an open-form qualitative report from home. Persisting psychological effects attributed to the psilocybin experience were measured using the Persisting Effects Questionnaire (PEQ) 3-months after administration. Using a linear latent-variable model we show that the MEQ total score is positively associated with the later emergence of positive PEQ effects (*p* = 3 × 10^−5^). Moreover, the MEQ subscales “Positive Mood” (*p*_corr_ = 4.1 × 10^−4^) and “Mysticality” (*p*_corr_ = 2.0 × 10^−4^) are associated with positive PEQ whereas the subscales “Transcendence of Time and Space” (*p*_corr_ = 0.38) and “Ineffability” (*p*_corr_ = 0.45) are not. Using natural language pre-processing, we provide the first qualitative descriptions of the “Complete Mystical Experience” induced by orally administered psilocybin in healthy volunteers, revealing themes such as a sense of connection with the Universe, familial love, and the experience of profound beauty. Combining qualitative and quantitative methods, this paper expands understanding of the acute psilocybin induced experience in healthy volunteers and suggests an importance of the type of experience in predicting lasting positive effects.

## Introduction

Psilocybin is a prodrug to the 5-HT2A agonist psilocin (4-hydroxy-N,N-dimethyltryptamine) (Passie et al., 2002), which is the drug responsible for the psychoactive effects of “magic mushrooms”. Preliminary evidence from the last 15 years suggest that psilocybin has a rapid and potent positive treatment effect in affective and addictive disorders (Moreno et al., 2006; Grob et al., 2011; Bogenschutz et al., 2015; Griffiths et al., 2016; Ross et al., 2016; Johnson et al., 2017; Carhart-Harris et al., 2018; Garcia-Romeu et al., 2019; Anderson et al., 2020; Davis et al., 2020; Carhart-Harris et al., 2021). In healthy individuals and patients, psilocybin has been associated with long-lasting positive psychological effects and self-reported positive changes in mood, behaviour (Griffiths et al., 2011; Barrett et al., 2020) and personality (e.g., increased openness; (MacLean et al., 2011; Erritzoe et al., 2018; Schmid and Liechti, 2018; Madsen et al., 2020; Kettner et al., 2021), while no association with persisting effects on cognition has been observed (Rucker et al., 2021).

The subjective effects of psychedelics vary between individuals (Stenbæk et al., 2020) and are substantially affected by dose, CYP2D6 phenotype (Vizeli et al., 2021) and non-pharmacological factors like emotional “set”, i.e., a person’s current psychological state, and environmental “setting”, i.e., the immediate surroundings (Hyde, 1960; Hartogsohn, 2017; Strickland et al., 2021). The acute effects of medium-high oral doses (i.e., >0.2 mg/kg) of psilocybin last approximately four to six hours (Passie et al., 2002; Hasler et al., 2004; Preller et al., 2018; Madsen et al., 2019; Stenbæk et al., 2020; Madsen et al., 2021) though psilocybin may produce a misjudged perception of time on behalf of the recipient of the drug (Wackermann et al., 2008). Subjective psychedelic effects are characterised by changes in bodily experience, cognition, and alterations in the visual field (Preller and Vollenweider, 2018; Hirschfeld and Schmidt, 2021), and by pseudohallucinations but not “true hallucinations” (sensory phenomena indistinguishable from reality) (Sanati, 2012). The typical experience induced by a medium-high dose of psilocybin has a plateau of maximum subjective intensity which, for some participants, can occasion transient, profound alterations in consciousness described as a sense of unity with all things accompanied by dissolution of ego or personhood, blissful mood and ecstasy, and aberrant sense of time and space (Preller and Vollenweider, 2018; Stenbæk et al., 2020). Despite extensive quantitative characterisation, no papers so far have reported a qualitative description of the subjective experience of orally administered psilocybin in healthy volunteers, though the effects of 2 mg of intravenous psilocybin in an MR scanning environment (Turton et al., 2014) and of 75 µg of oral LSD (Sanz et al., 2021) have been described previously.

At maximum intensity psychedelic experiences can share many similar features with traditional “mystical-type” or “peak’ experiences which have a potential for psychological transformation (Maslow, 1959; Griffiths et al., 2006; Griffiths et al., 2011; Lyvers and Meester, 2012). A subset of these, known as “Complete Mystical Experiences” (CME) are hypothesised to be instrumental in the lasting positive effects of psychedelics (Yaden and Griffiths, 2021). The magnitude of psilocybin-induced mystical-type experience has shown positive correlations with improvements in subjective life quality, meaning in life, and mood in patients suffering from anxiety and depression associated with life threatening cancer (Griffiths et al., 2016; Ross et al., 2016), cigarette addiction (Garcia-Romeu et al., 2015) and major depressive disorder (Roseman et al., 2018; Davis et al., 2020). In healthy volunteers, the degree of mystical experience has been positively associated with increases in personality-domain openness up to 14-months after psilocybin (Griffiths et al., 2008; MacLean et al., 2011; Griffiths et al., 2018) and self-rated persisting positive effects 12-months after psilocybin (Schmid and Liechti, 2018). Despite replicated research investigating the acute effects of psychedelics, comparatively little is known about the relation between the character of the acute psychedelic experience (i.e., the way the experience feels, not simply the intensity) and the persisting changes in mood, behaviour, and personality.

Qualitative research so far has provided insight into themes that patients report as important in the lasting effects of psychedelic therapy including increased aesthetic appreciation and experiences of interconnectedness (Noorani et al., 2018), movement from disconnection to connection with senses, self, others and the world, and from emotional avoidance or numbness to emotional acceptance (Watts et al., 2017). In terminally ill patients, themes observed to be associated with clinical improvement include reconciliation with death and reconnection with life (Swift et al., 2017). Patients’ qualitative reports also describe meaningful aspects of the acute experience including altered perception of relationships and the appearance of meaningful visual phenomena (Belser et al., 2017) as well as acutely felt motivation and commitment to behavioural change (Nielson et al., 2018). However, qualitative analyses are particularly susceptible to investigator bias, as quote selection can be driven by the investigator’s proposed narrative (Galdas, 2017). We aimed to control for this for by first pre-analysing data using an unbiased natural language processing (NLP) script to steer qualitative analyses.

In this study, we contribute to the ongoing research concerning the relation between the acute subjective effects of psilocybin and their lasting effects. We do so by examining whether the self-reported intensity of the mystical-type experience is associated with persisting effects reported 3 months later in healthy volunteers. Further, we examined whether NLP of qualitative reports can be used to differentiate participants who have a CME compared to those who do not. We also expand the literature describing the subjective mystical-type experience induced by psilocybin by providing curated quotes from the qualitative reports guided by NLP results.

We hypothesise that there will be a positive association between intensity of the mystical-type experience and persisting positive effects attributed to the psilocybin experience at 3-month follow-up. In addition, we provide NLP of qualitative reports to describe the experiences of participants who have a CME compared to those who do not.

## Methods

### Participants

#### Recruitment

We recruited 28 healthy participants (12 females) ≥18 years of age from a list of individuals that expressed interest in participating in a psilocybin neuroscience study. Individuals participated in at least one of three sub-projects (sub-project 1 (*n* = 4), sub-project 2 (*n* = 10) and sub-project 3 (*n* = 21)) for a total of 35 psilocybin administrations (i.e., seven participants took part in two sub-projects). The sample included in this study only included those who had completed the Qualitative Experience Report as described below, and received at least 12 mg of psilocybin.

All participants underwent screening for somatic illness, including a medical examination, an ECG, blood screening for somatic disease, and screening for psychiatric disorders using the Mini International Neuropsychiatric Interview, Danish translation version 6.0.0 (Sheehan et al., 1998). Exclusion criteria for all sub-projects were: (Passie et al., 2002) present or previous primary psychiatric disease (DSM axis 1 or WHO ICD-10 diagnostic classifications) or in first-degree relatives; (Carhart-Harris et al., 2018) present or previous neurological condition/disease, significant somatic condition/disease; (Carhart-Harris et al., 2021) intake of drugs suspected to influence test results; (Davis et al., 2020) non-fluent Danish language skills; (Moreno et al., 2006) vision or hearing impairment; (Bogenschutz et al., 2015) previous or present learning disability; (Garcia-Romeu et al., 2019) pregnancy; (Johnson et al., 2017) breastfeeding; (Griffiths et al., 2016) magnetic resonance imaging (MRI) contraindications; (Grob et al., 2011) alcohol or drug abuse; (Ross et al., 2016) allergy to test drugs; (Anderson et al., 2020) significant exposure to radiation within the past year (e.g., medical imaging investigations); (Barrett et al., 2020) intake of QT-prolonging medication or electrocardiogram (ECG) results indicative of heart disease (Griffiths et al., 2011), blood donation less than 3 months before project participation; (MacLean et al., 2011) bodyweight less than 50 kg; (Erritzoe et al., 2018) low plasma ferritin levels (<12 μg/L).

#### Ethics

Written informed consent was obtained from all individuals before inclusion. The study was conducted in accordance with the Declaration of Helsinki. The study was approved by the ethics committee for the Capital Region of Copenhagen (journal identifier: H-16028698, amendments: 56,023, 56,967, 57,974, 59,673, 60,437, 62,255, and Danish Medicines Agency (EudraCT identifier: 2016-004000-61, amendments: 2017014166, 2017082837, and 2018023295). The study was also preregistered at ClinicalTrials.gov (identifier: NCT03289949).

### Psilocybin Intervention

Prior to the intervention day, all participants attended a preparatory consultation with the study psychologists who would assist them on the intervention day. In all three sub-projects, psilocybin was administered orally in 3 mg capsules with a glass of water. Dose was adjusted for bodyweight: mean (SD) [range] = 0.26 (0.04) [0.19–0.31] mg/kg, absolute dose: mean (SD) [range] = 19.4 (3.7) [12–30] mg. Interpersonal support was provided throughout the interventions in all three sub-projects by the same leading psychologist (DSS) assisted by psychologist trainees. Likewise, the same medical doctor was involved in all three sub-projects (MKM). A light lunch and beverages (i.e., water + juice) were offered to the participants on all intervention days. Among the 35 sessions, four were conducted in the context of up to two positron emission tomography (PET) scans on the intervention day in sub-project 1 (Madsen et al., 2019), 10 sessions were conducted in a comfortable hotel-room-like setting in sub-project 2 (Madsen et al., 2020; Stenbæk et al., 2020), and 21 were conducted in the context of MRI scans on the intervention day in sub-project 3 (Madsen et al., 2021). All participants returned 1 day after the intervention day for a post-session consultation with the assisting psychologists.

### Data Collection and Outcome Measures

#### Baseline Psychometrics

The outcome measures used in this study consisted of qualitative and questionnaire data collected on the day of psilocybin intervention and at 3-month follow-up. At baseline, we also collected descriptive information about depressive symptoms, perceived stress and sleep quality with the Major Depression Inventory (MDI) (Bech et al., 2001), Cohen’s Perceived Stress Scale (PSS) (Cohen et al., 1983) and Pittsburgh Sleep Quality Index (PSQI) (Buysse et al., 1989), respectively.

#### Mystical Experience Questionnaire

At the end of the psilocybin intervention (∼6 to 8 hours after ingestion of psilocybin), participants completed the self-report revised 30-item Mystical Experience Questionnaire (MEQ) (Barrett et al., 2015). The MEQ is a validated questionnaire comprising 30 items that capture the essential elements of a mystical-type experience relating to a single, discrete event (e.g., one psilocybin administration). The MEQ was derived from the MEQ43 which in turn was developed from the States of Consciousness Questionnaire based on classical descriptive work on mystical experiences and the psychology of religion (Stace, 1960; Pahnke, 1969; Richards, 1975). Items in the MEQ are rated using a 6-point Likert scale from 0 (none, not at all) to 5 (extreme, more than any other time in my life and more than a rating of 4). It has four sub-scales: Mysticality, Positive Mood, Transcendence of Time and Space and Ineffability (e.g., MEQ items: “Experience of unity with ultimate reality”, “Experience of ecstasy”, “Loss of your usual sense of time” and “Sense that the experience cannot be described adequately in words”, respectively). The threshold for a CME is a score of >60% on all sub-scales (Barrett et al., 2015). This threshold was used to divide data into CME and non-CME. MEQ total score was calculated as the mean of all items.

#### Mandala Drawing

Following completion of the MEQ, participants were asked to draw a mandala of their experience using coloured pencils. Mandala is the Sanskrit word for “circle”, and it is typically interpreted as symbolising a transcendent function in the psyche (Bailer and Leeming, 2020). Practically, participants were given a paper with a circle represented on it and asked to draw their psilocybin experience within the circle using coloured pencils.

#### Qualitative Experience Report

In the evening on the day of psilocybin intervention, participants completed a qualitative report describing their experience in free writing using a secured online survey system (https://www.limesurvey.org/). There were no constrains and no minimum or maximum length to the experience report, only that it had to be completed before the participants went to sleep.

#### Persisting Effects Questionnaire

Approximately 3 months following the psilocybin sessions, participants completed the Persisting Effects Questionnaire (PEQ) (Griffiths et al., 2006). The PEQ is used to measure long-lasting positive and negative effects attributed directly to the psychedelic experience. The PEQ is a non-validated self-report questionnaire that has been used, among others, by Griffiths et al. (2006), Griffiths et al. (2008), Griffiths et al. (2011), Griffiths et al. (2016) to assess persisting effects of psychedelics as well as long-term positive effects attributable to “naturally occurring” mystical-type experiences (Griffiths et al., 2019). The PEQ comprises 145 questions that describe how the participants perceive changes in their life that they feel are “due to the experiences during your last session [of psilocybin] and your contemplation of those experiences”. Items are rated using a 6-point Likert scale from 0 (none, not at all) to 5 (extreme, more than any other time in my life and more than a rating of 4). It has 12 subscale-themes, namely a positive and a negative change in: Attitudes About Life, Attitudes About Self, Mood Changes, Social Effects, Behavioural Changes, and Spirituality (e.g., Attitudes about life: “You have more joy in your life” and “You have less joy in your life”, Social Effects: “You express more love toward others” and “You express more hatred toward others”). Subscale scores were converted to percentage of maximum score as performed in previous analyses (Griffiths et al., 2011; Griffiths et al., 2016). For the statistical analyses within study, we used the positive subscales only since very few persisting negative effects were rated. See Figure 2.

#### Plasma Psilocin Levels

In sub-project 3 plasma samples were drawn before and approximately 40, 80, 110, 160, 190, and 340 min post-psilocybin administration, and plasma psilocin levels (PPL) were evaluated as described in (Madsen et al., 2019). Peak values were chosen as the MEQ specifies “at any time during that session you experienced the following phenomena”.

### Statistical Analyses

#### Descriptive Analyses

Differences at baseline between those who experienced a CME and did not experience a CME (non-CME) on the MDI, PSS, PSQI, age differences, bodyweight, dose administered, and naivety to psychedelic experiences were compared using a random intercept model to take into account that some participants participated twice. Sex differences in MEQ subscale and total score were also estimated using a similar model. Associations between the highest PPL value, i.e., representing peak effects of the drug, for each participant and MEQ total score or PPE_LV_ were evaluated using univariate linear models. There were no repeated observations in this subgroup.

#### Association Between MEQ and PEQ

**FIGURE 1:**
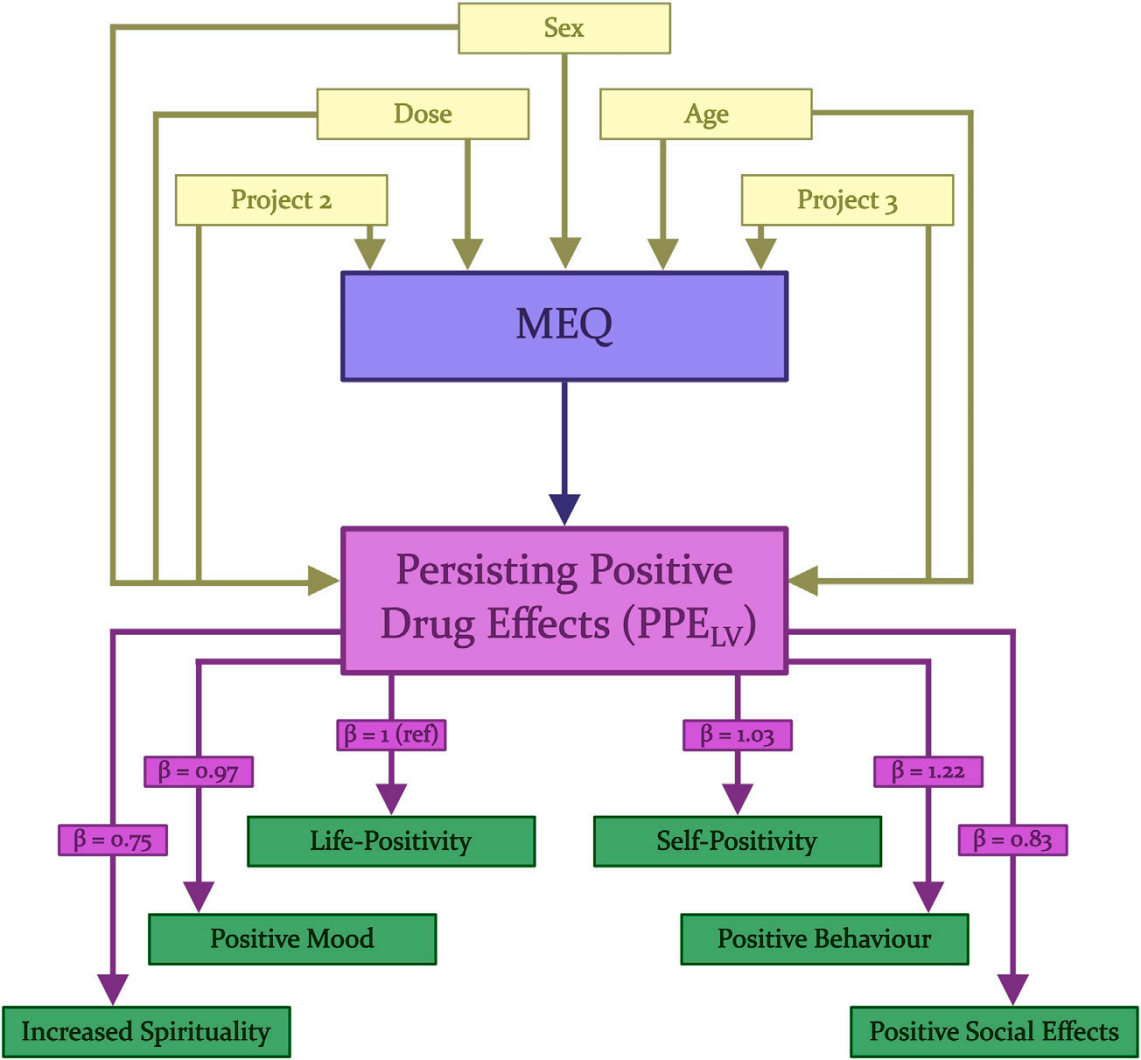
Latent Variable Model. Schematic diagram describing the structure of the LVM used in the analysis of the relation between MEQ and the latent variable PPE_LV_. “MEQ” (blue) represents either the MEQ total score or one of the four different subscales of the MEQ. Each was analysed using a different model; therefore, regression outputs are not reported in this figure and are instead reported in Table 2. Loadings of the individual positive subscales of the PEQ are stable between models and are thus reported on the lines between PPE_LV_ and the subscales. Loadings of co-variates are reported in Supplementary Table S3.

The association between the MEQ and the PEQ, was investigated with linear latent variable models (LVMs) using the “lava” package for R (Holst and Budtz-Jørgensen, 2013). LVMs can be seen as an extension of linear mixed models for the analysis of multiple repeated measurements. While linear models parameterised with a random slope assume a constant correlation between measurements, LVMs relax this assumption allowing certain measurements to be more correlated than others (i.e., measurements from certain subscales can be more intercorrelated than others). LVMs were evaluated using the “modelsearch” function from the lava package and typically structured as shown in Figure 1 with some variation in covariance as described in section *Latent-Variable Model Construction*. A latent variable representing the six subscales of the PEQ was constructed and termed Positive Persisting Effects (PPE_LV_). The association between the MEQ total and PPE_LV_ was evaluated within this model. Post hoc analyses of the associations between MEQ subscale scores and PPE_LV_ were also evaluated, each in a separate model. In order to calculate the potential direct and indirect mediation effects of the co-variates “Age”, “Sex”, “Dose”, “Project 2” and “Project 3”, both direct regressions of these onto PPE_LV_ and indirect effects of these via MEQ total or subscales were calculated in each model. “Sex-Female” and “Project 1” are reported as the intercept in the analysis of these co-variates. We report loadings (i.e., parameter for the association subscale-latent variable, denoted by “β” in Figure 1) and confidence intervals of each PEQ subscale onto PPE_LV_ with “life-positivity” arbitrarily chosen as a reference. To control for the small sample size and repeated measures, robust standard error was used (Ozenne et al., 2020). The MEQ total model was evaluated first as a primary hypothesis and the *p*-value for the relation between the MEQ total score and PPE_LV_ is reported. Subsequent tests investigating the subscales report the *p*-value for the same interaction in the model, corrected using the Bonferroni method for a family of four tests (Dunn, 1961).

### Language Analyses

#### Lemmatisation

All evaluations were performed on the untranslated Danish text. Instances of words and quotes presented in this paper were independently translated by two bilingual individuals and recombined by a native English speaker and a third bilingual person.

In order to pre-process the qualitative data for this analysis, words were lemmatised using the python package “lemmy” v 2.1.0 (https://github.com/sorenlind/lemmy). Lemmatisation is the process of converting a word back to its root (i.e., “running” “ran” and “runs” would each be lemmatised to “run”). Danish words are not easily lemmatised as any given word may represent different cases for several different roots. For example, “slappe” (relax) could be lemmatised to “slappe” (relax), “slap” (loose), or “slippe” (drop). In order to control for this, the first lemma for each word was selected and others discarded. Upon random manual checking, this was accurate in all instances.

#### Tf-idf Analyses

In this paper we attempt to control for investigator-narrative bias by utilising term-frequency inverse document frequency (tf-idf) to identify distinguishing themes in the text prior to qualitative analysis (Ramos, 2003). The corpus of all reports was split into two documents containing reports from either CME or non-CME. Tf-idf is a statistic that reflects the importance of a term to a specific subset of documents within a wider corpus. Term frequency is the number of times the word appears in each document divided by the total number of words in that document. Inverse document frequency is the log of the total number of documents divided by the number of documents containing the word, and tf-idf is the product of these. Reports were pre-processed by lemmatisation as described above, but stop-words (e.g., “the”, “if”, “and” etc.) were not removed as the tf-idf process accounts for these. Tf-idf scores were computed for each term within CME and non-CME documents. Analyses were performed using the R package “tidytext” (Silge and Robinson, 2016).

$$tf-idf=\frac{incidence\;of\;x\;in\;document}{total\;length\;of\;document}\times log_{10}\left(\frac{total\;number\;of\;documents}{number\;of\;documents\;containing\;x}\right)$$

#### Post-hoc Qualitative Analyses

To further investigate the terms with the highest tf-idf scores, we manually searched through all reports and extracted quotes that included each of the five highest tf-idf terms. We first checked that each term was not driven by a single report that used the word repeatedly. We then selected quotes that reflected the most representative thematic usages of these terms.

#### Mandala Assignment

To further illustrate our qualitative findings, available mandala drawings were located that aligned with the qualitative reports presented. Those interpreted as aligning with the themes of the associated qualitative report were selected, and the five participants were contacted to ensure that perception of depicted themes was accurate. These are presented in the results alongside their respective quotes.

### Code Availability

R and python scripts used in analyses are available at https://github.com/Pneumaethylamine/MysticalPEQ.

### Statistical Inference

Throughout the paper statistical significance was considered at an alpha of *p* < 0.05 following Bonferroni correction for multiple comparisons where appropriate (Haynes, 2013). We report corrected and uncorrected *p*-values, estimates and 95% confidence intervals.

## Results

### Descriptive Analyses

**FIGURE 2:**
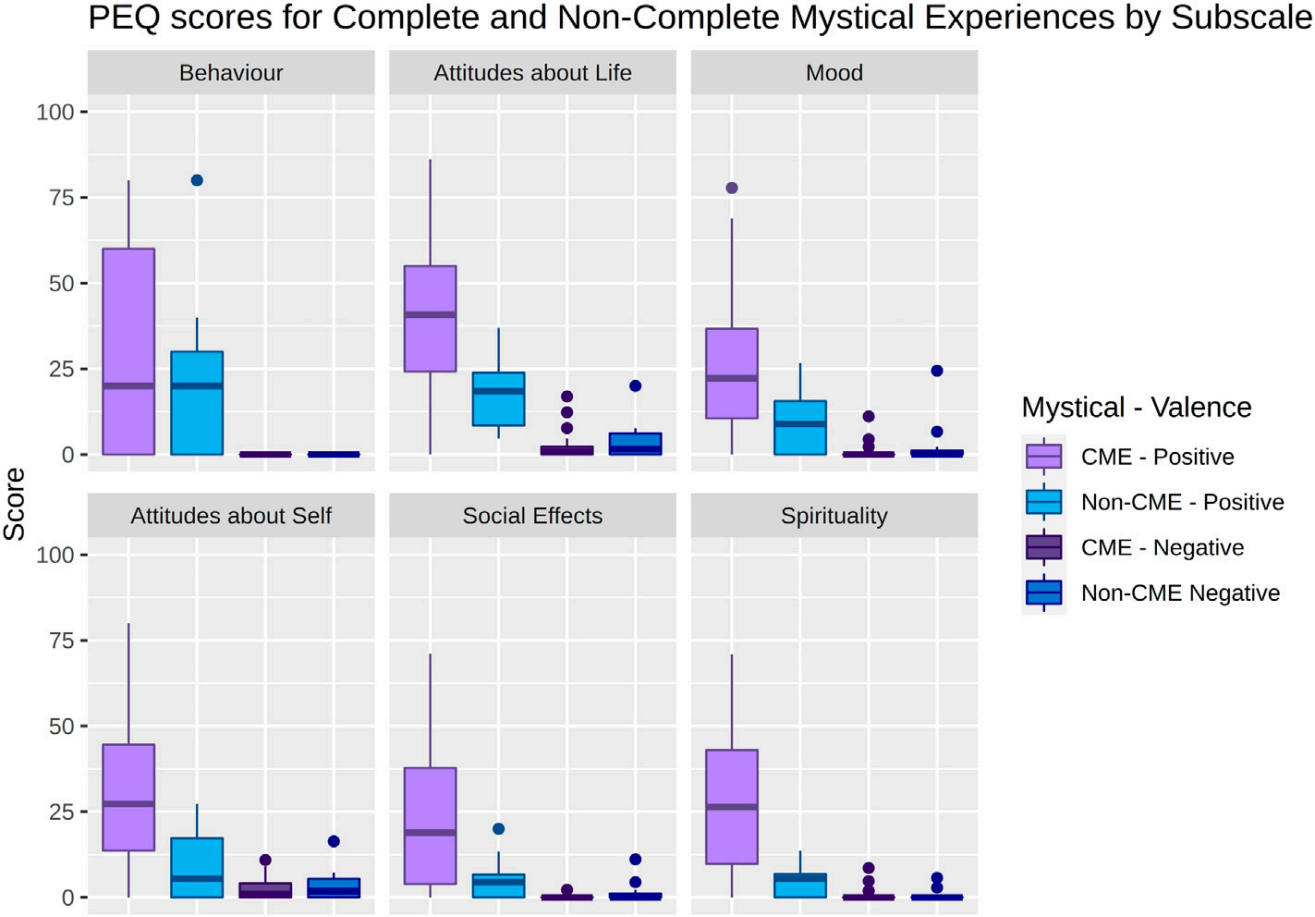
Boxplots showing Persisting Effect Questionnaire (PEQ) scores relating to either Complete Mystical Experiences (CME) (Purple) or non-CME (Blue). Each plot represents the positive (lighter colours, left) and negative (darker colours, right) aspects of each PEQ subscale. The middle line in each box represents the median. Lower and upper hinges represent first and third quartiles. Datapoints more than 1.5 interquartile range beyond the hinges are plotted as outliers.

In total 35 reports from 28 individual participants were recorded as seven participants took part in two projects. Of the 35 reports, the mean age (SD) was 31.7 (7.0) [range = 24.3 to 58.8] years, and 15 were from female subjects. One participant had missing PEQ data at 3-month follow-up. Therefore, this dataset was not included in the analyses involving the PEQ. Individual doses, sex, MEQ total and subscale scores are reported in Supplementary Table S1. Descriptive statistics of participants, psilocybin sessions, project, sex, age, weight, naivety to psychedelics, dose, and baseline psychometrics for all participants combined and split by CME and non-CME are shown in Table 1, indicating no significant differences in these metrics between groups. PEQ subscale scores split by CME are presented in Figure 2. MEQ total and subscale scores are reported in Supplementary Table S2. A linear mixed effects model comparing MEQ total score and subscales between males and females, accounting for participant as a random variable and treating “Female” as the intercept showed no significant sex difference in any score (p_corr_ > 0.31). A description of the direct and indirect mediation effects of sex and other co-variates on PPE_LV_ can be found in Supplementary Table S3. No relation was observed between peak PPL and MEQ total score (*p* = 0.88, uncorrected) or PPE_LV_ (*p* = 0.35, uncorrected). See Supplementary Figure S2.

**TABLE 1:** Descriptive statistics of the participants split by Complete Mystical Experience (CME).

Measure	All	CME	Non-CME	*p* value
Participants (no.)	28	16*	12*	NA
Psilocybin sessions (no.)	35	21	14	NA
Project 1 (PET) (no.)	4	1	3	0.31
Project 2 (no.)	10	7	3	0.21
Project 3 (MRI) (no.)	21	13	8	0.28
Female (%)	15 (43%)	8 (38%)	7 (50%)	0.50
Mean age (SD) (years)	31.7 (7.0)	30.8 (4.9)	33.1 (9.4)	0.45
Mean weight (SD) (kg)	72.4 (12.2)	75.5 (11.9)	72.7 (12.7)	0.44
Psychedelic Naïve (no. (%))	23 (66%)	14 (67%)	9 (64%)	0.89
Mean dose (SD) (mg)	19.4 (3.7)	19.3 (3.5)	19.5 (4.2)	0.80
Mean dose (SD) (mg/kg)	0.26 (0.04)	0.26 (0.04)	0.27 (0.04)	0.34
Mean baseline MDI	4.2 (2.4)	4.1 (2.6)	4.3 (2.2)	0.94
Mean baseline PSS	6.8 (3.8)	6.7 (3.6)	7.1 (4.1)	0.70
Mean baseline PSQI	3.7 (1.7)	3.3 (1.5)	4.3 (1.9)	0.18

All descriptive statistics are mean (Standard Deviation) unless otherwise stated. These describe the baseline characteristics including Major Depressive Inventory (MDI), Perceived Stress Scale (PSS), and Pittsburgh Sleep Quality Index (PSQI), aspects of the acute experience including dose of psilocybin administered (*per os*) and subproject. *p*-values for projects were calculated using a chi-squared test. All other *p*-values were calculated using a linear mixed-effects model controlling for participant ID as a random variable. PET, Positron Emission Tomography; MRI, Magnetic Resonance Imaging. *All individuals observed twice had either a CME or non-CME both times.

### Association Between MEQ and PEQ

#### Latent-Variable Model Construction

All six positive subscales of the PEQ loaded well onto the latent variable PPE_LV_ (Estimate range = 0.75:1.22, *p* < 1.1 × 10^−10^ for all subscales). All subscale loadings for each model are reported in Supplementary Table S4. No additional covariances were modelled in the main analysis evaluating MEQ total. The models for subscales Ineffability, Transcendence of Time and Space, and Mysticality contained covariance between the PEQ subscales “Positive Attitudes about Life” and “Positive Attitudes about Self” whereas the model investigating the Positive Mood subscale contains a covariance between the MEQ subscale Positive Mood and the PEQ subscale “Increased Spirituality”.

#### Association Between PEQ and MEQ

**FIGURE 3:**
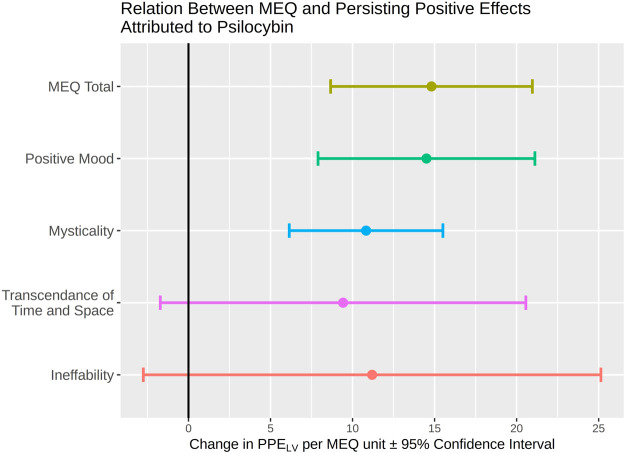
Results of the latent variable model describing the estimate of effect of the MEQ total and subscale scores measured on the day of psilocybin on PPE_LV_ measured 3 months after psilocybin. The *y*-axis represents each individual subscale while the *x*-axis provides an estimate of the difference in PPE_LV_ associated with a one-unit increase in the indicated MEQ subscale.

The LVM investigating the association between MEQ total score and PPE_LV_ showed a statistically significant positive association (*ß* = 14.8, 95%CI = 8.66:20.96, *p* = 3 × 10^−5^). The subsequent LVMs investigating effects of the MEQ subscales on PPE_LV_ showed significant positive association with Positive Mood (*ß* = 14.5, 95%CI = 7.90:21.11, p_corr_ = 4.1 × 10^−4^), as did the LVM evaluating Mysticality (*ß* = 10.8, 95%CI = 6.14:15.51, p_corr_ = 2.0 × 10^−4^). However, no significant associations were observed for Transcendence of Time and Space (*ß* = 9.4, 95% CI = −1.72:20.56, p_corr_ = 0.38) or Ineffability (*ß* = 11.2, 95% CI = −2.75:25.14, p_corr_ = 0.45). See Table 2 for a full summary of the LVM results and Figure 3 for a graphical representation. LVM model summaries are reported in Supplementary Table S4. Direct and indirect mediation effects of covariates are reported in Supplementary Table S3. No indirect effects of any covariate via MEQ were significant (all *p* > 0.1, uncorrected). Scatter plots of MEQ total and subscales by PPE_LV_ are reported in Supplementary Figure S1.

**TABLE 2:** Results from the Latent Variable Model analyses for Mystical Experience Questionnaire (MEQ) total score and subsequent subscale analyses.

Variable	*ß*	95% CI	*p*-value	*p*-value (corrected)
MEQ total	14.8	8.66:20.96	3.0 × 10^−5^	3.0 × 10^−5^
Positive Mood	14.5	7.90:21.11	1.0 × 10^−4^	4.1 × 10^−4^
Mysticality	10.8	6.14:15.51	5.1 × 10^−5^	2.0 × 10^−4^
Transcendence of Time and Space	9.4	−1.72:20.56	0.094	0.377
Ineffability	11.2	−2.75:25.14	0.111	0.446

*ß* and 95% CI indicate effect size and 95% confidence interval in units of the latent variable PPE_LV_ which represents persisting positive effects attributable to the drug experience as a % of maximum possible score.

### Language Differences in Mystical and Non-Mystical Experiences

#### Natural Language Processing

**FIGURE 4:**
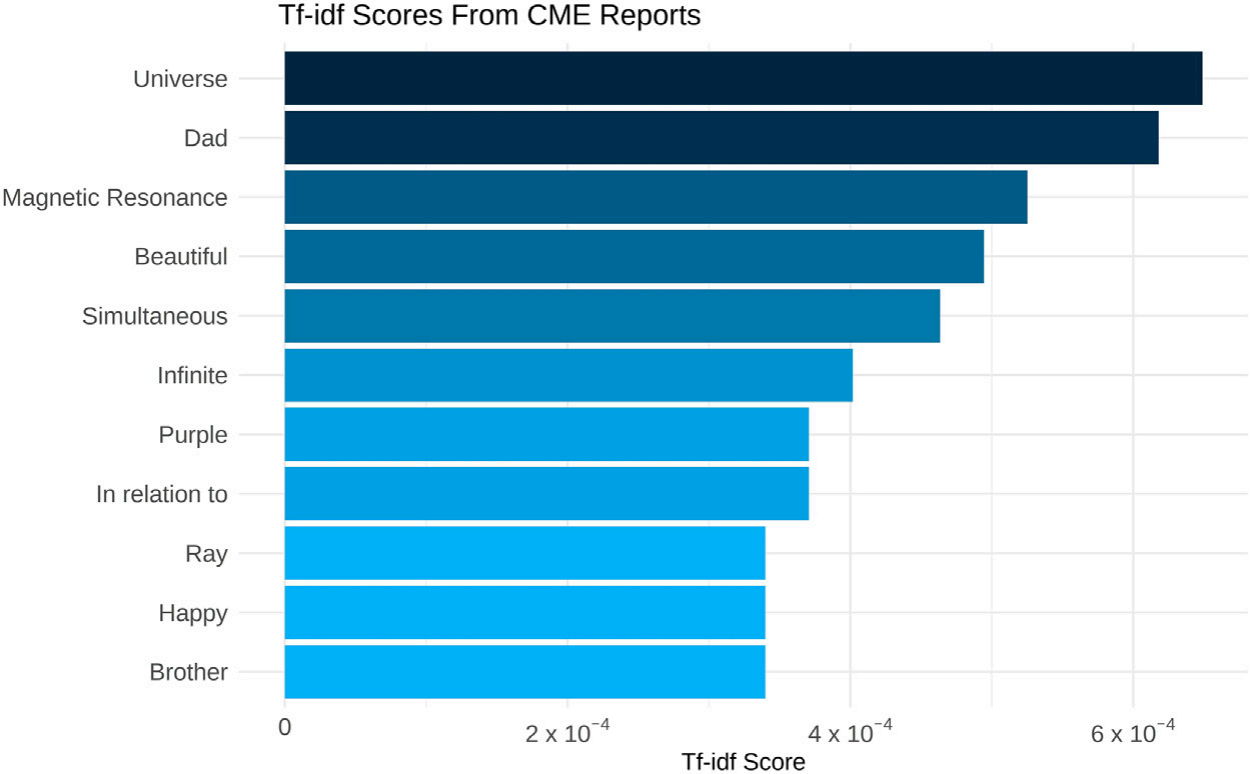
Bar chart displaying the term-frequency inverse document frequency (tf-idf) values for the words that only appear in “Complete Mystical Experience” (CME) reports. Analyses were performed on Danish words and are only translated into English for display in this figure.

Of the 35 reports analysed, 21 met the criteria for a CME i.e., scoring at least 60% on all subscales of the MEQ. The words with the highest tf-idf score from CME Qualitative Experience Reports indicating the greatest distinction from the overall corpus, were “Universe”, “dad”, “MR” (Magnetic Resonance), “beautiful”, “simultaneous”, “infinite”, “purple”, “in relation to”, “ray”, “happy”, and “brother” (See Figure 4). The highest tf-idf scores from non-CME reports were “gloomy”, “cycle”, “evil”, “cold” and “need”. To expand on these findings, these words were identified in text and a selection of quotes are presented below. Notably, as there are only two documents within the corpus (CME and non-CME), any word which appears in both documents has a tf-idf score of zero. Thus, tf-idf scores distinguish words that only appear in one document set (CME or non-CME).

#### Qualitative Reports of the Complete Mystical Experience

By manually searching through the document body, we confirmed that high tf-idf scores were not driven by single individuals utilising the identified word multiple times. The word with the highest tf-idf score from the CME reports was “Universe”. Viewed in context, these references to the Universe appear to relate to feelings of unity and connectedness, analogous to MEQ item 14 “Freedom from the limitations of your personal self and feeling a unity or bond with what was felt to be greater than your personal self”, as well as a wonderment around the complexity of conscious experience.

*The trip was mostly concerned with the love pertaining to the different relations in my life, but also the love that exists between human beings in general, to the planet and to the universe.*

*Report 3, female, CME, MEQ total 3.9*

*The light of love brings clarity to everything. I get a deep feeling of purity and feel that everything is beautiful, and that love is what makes up the world and the universe and connects everything like a network of roots.*

*Report 22, female, CME, MEQ total 3.9*

*Several times during the trip, I found myself laughing in sheer admiration of the impressive and wonderous universe of human consciousness.*

*Report 10, male, CME, MEQ total 4.7*

*When I close my eyes, I am in a fantastic world. Suspended in the whole universe. Stretched across the cosmos. When I open my eyes, I am being prepared for the MR-scanner. […] I can hear the sounds of the scanner which influence my universe, white and silver lines unfold, make sudden angles and travel onwards abruptly.*

*Report 31, male, CME, MEQ total 4.2*

One report juxtaposes the profound noetic quality of the experience with the tasks that participants were asked to perform in the scanner environment. This aligns with item 9 of the MEQ, i.e., “Certainty of encounter with ultimate reality (in the sense of being able to “know” and “see” what is really real at some point during your experience).”

*I have the answer to the riddle of the universe, but I’m forced to look into a TV-screen.*

*Report 31, male, CME, MEQ total 4.2*

The word that next-best distinguished the CME was “dad”. The reports describe feelings of gratitude, love and respect for the participants’ fathers. There are no items of the MEQ that reflect this theme directly. Notably, references to the participants mothers appeared in both CME and non-CME reports and thus had a tf-idf score of zero.

*I had the feeling that I was experiencing the world through myself as a little girl holding her dad’s hand. My dad and I were observing what was happening around us. I think we saw something that resembled beautiful nature and charming castles.*

**FIGURE 5:**
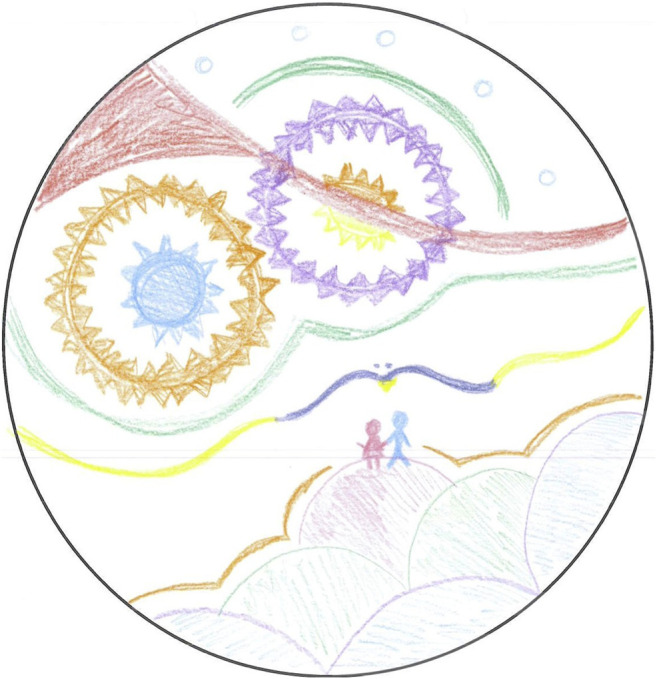
I had the feeling that I was experiencing the world through myself as a little girl holding her dad’s hand. My dad and I were observing what was happening around us. I think we saw something that resembled beautiful nature and charming castles. Report 6, female, CME, MEQ total 3.9

*Report 6, female, CME, MEQ total 3.9*, see Figure 5

*My mum and dad are opposites, red and white, cold and warm, but they are like a perfect yin-yang melting together, like my drawing shows, and their dynamic dance of east and west creates a space in the middle where it is purple—I am purple—I am both of them.*

*Report 14, female, CME, MEQ total 4.5*

In two cases, the participants describe a desire to share this experience with their fathers specifically.

*The tears run down my cheeks as I feel an enormous sense of closeness and connectedness. I feel an enormous sense of love towards my dad, his way of being and his patience. I want my dad to experience the feeling of ultimate love as I do, and for him to have the same experience. I wish to share my love with him.*

*Report 12, male, CME, MEQ total 4.9*

The word with the third highest tf-idf score was “MR” referring to the MRI scanner. In several cases, participants found the intense noises of the scanner influenced their experience, in some cases inducing synaesthesia-like experiences. More broadly, this represents the profound impact of the environment on the experience.

*The sounds from the MR scanner each cast a specific hue, with high-frequency tones giving a yellowish tinge and low-frequency tones having a purple tinge.*

*Report 22, female, CME, MEQ total 3.9*

The MR environment was the setting for 21 out of the 35 experiences collected (sub-project 3). Some felt that it played a positive role in the experience, as described in item 6 of the MEQ “Experience of oneness or unity with objects and/or persons perceived in your surroundings.”

*I felt a sense of no longer being connected to my own body. The MRI scanner and I stepped into a different reality together. With colours, and shapes, and figures.*

**FIGURE 6:**
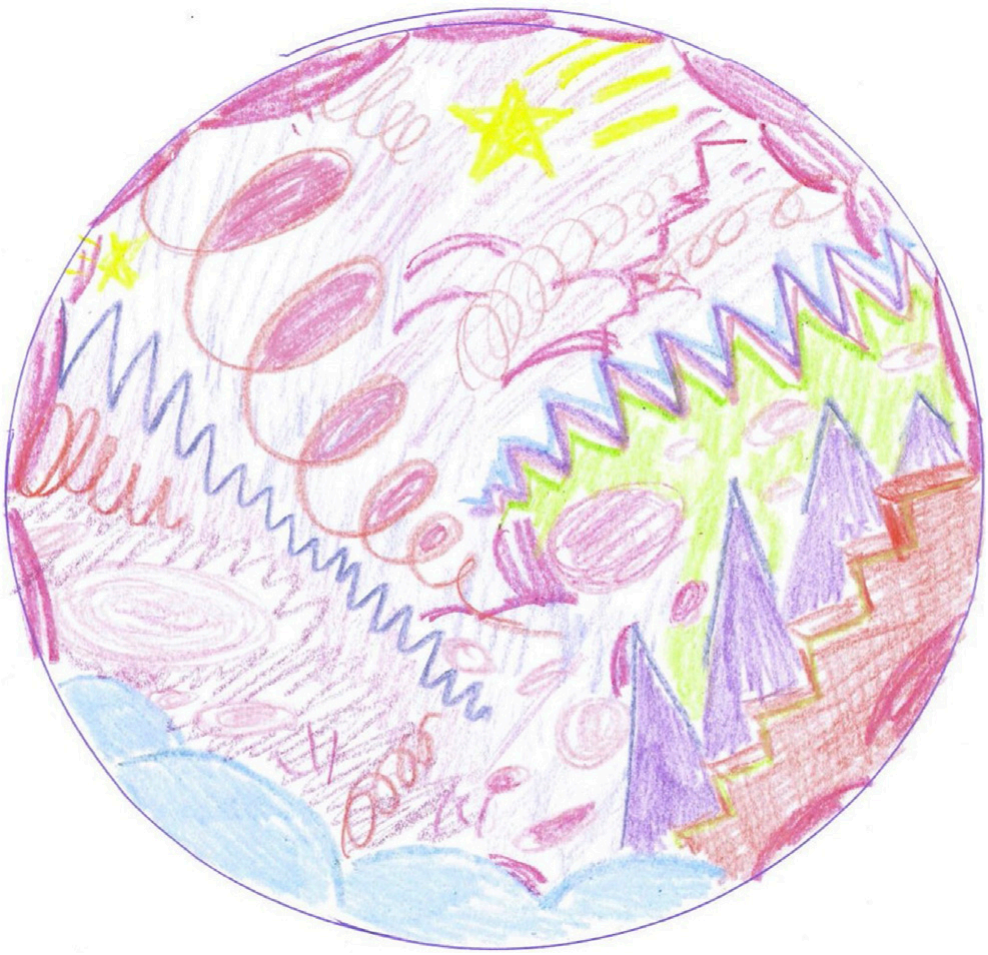
I felt a sense of no longer being connected to my own body. The MRI scanner and I stepped into a different reality together. With colours, and shapes, and figures. Report 32, female, CME, MEQ total 4.7.

*Report 32, female, CME, MEQ total 4.7*, see Figure 6

The word with the fourth highest score was “beautiful”. This particular excerpt below combines the themes of universality, family and beauty that appear repeatedly in the CME reports. This report also aligns with item 8 in the MEQ “Feelings of tenderness and gentleness”.

*The feeling of joy and love was the energy in the universe, completely intense and multiplied by 100. It was like taking those two emotions and concentrating them, to have them in their purest form without any worries or other troubles that can come with everyday reality. Everything else ceased to matter while those two emotions were so pure—they made everything incredibly beautiful. When I was soaring through the soundwaves with this energy, I could see all the people close to me in my life appear. My partner, my sister and her boyfriend and their new-born son, my mother, my amazing friends. I felt immensely privileged to be part of this universe / community, to be able to feel those feelings in such a pure form, to have such deep emotions in my body—and I was overwhelmed with gratitude for this world. That everything simply is. And with that I was overtaken by a desire to protect it all, to show the world how beautiful it is and to take care of it.*

**FIGURE 7:**
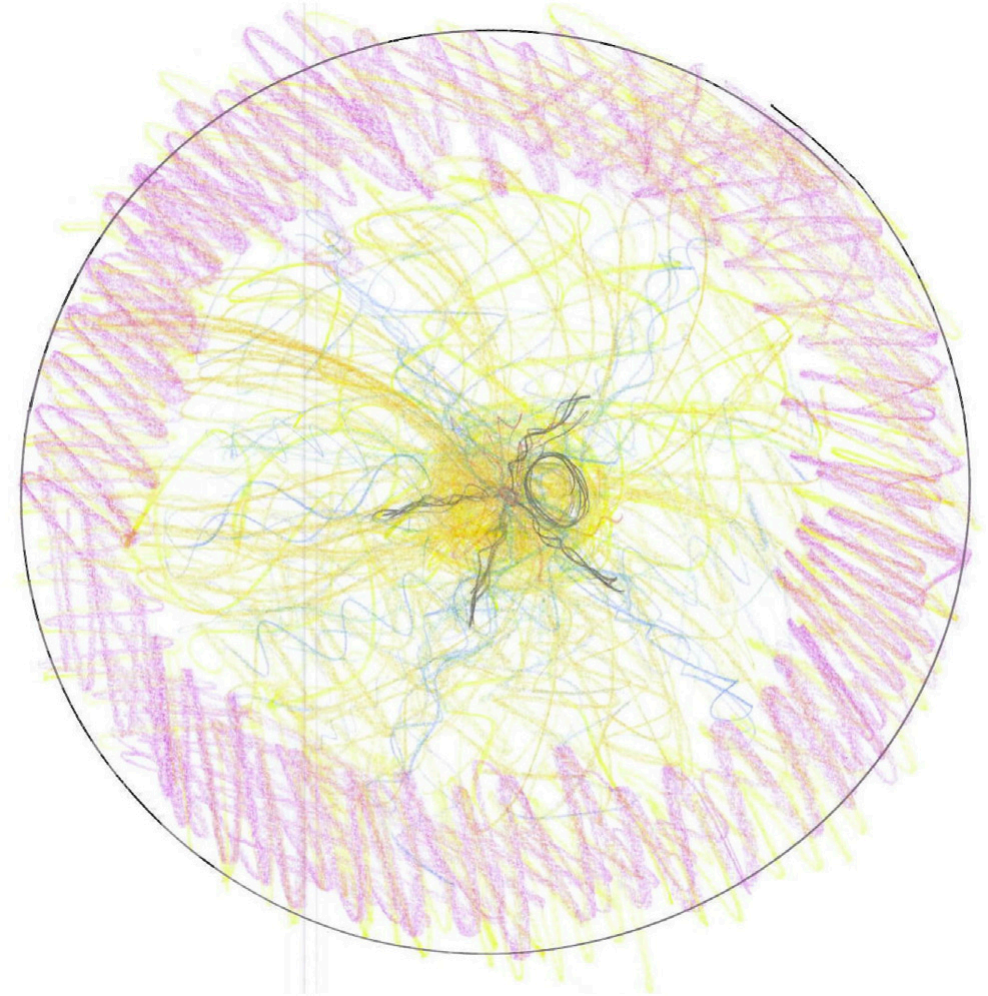
The feeling of joy and love was the energy in the universe, completely intense and multiplied by 100. It was like taking those two emotions and concentrating them, to have them in their purest form without any worries or other troubles that can come with everyday reality. Everything else ceased to matter while those two emotions were so pure—they made everything incredibly beautiful. When I was soaring through the soundwaves with this energy, I could see all the people close to me in my life appear. My partner, my sister and her boyfriend and their new-born son, my mother, my amazing friends. I felt immensely privileged to be part of this universe/community, to be able to feel those feelings in such a pure form, to have such deep emotions in my body—and I was overwhelmed with gratitude for this world. That everything simply is. And with that I was overtaken by a desire to protect it all, to show the world how beautiful it is and to take care of it. Report 3, female, CME, MEQ total 3.9.

*Report 3, female, CME, MEQ total 3.9*, see Figure 7

Several reports refer to the beauty of the closed eye visual effects and the participants interactions with them. Perceptual effects are not measured by the MEQ.

*Beautiful, beautiful geometrical shapes—and I spin around, do backflips—for a moment I just enjoy doing summersaults. My body diffuses into rays of sand/light and disappear, but as soon as they’re completely gone and I think to myself “oh, all gone, oh well”, they remanifest themselves into a new meaningful picture.*

*Report 14, female, CME, MEQ total 4.5*

*My inner vision became the universe, filled with colourful waterfalls, glistening like stars, not bound by gravity. They floated in the air and folded around each other. Totally quiet. The streams were infinite and beautiful.*

**FIGURE 8:**
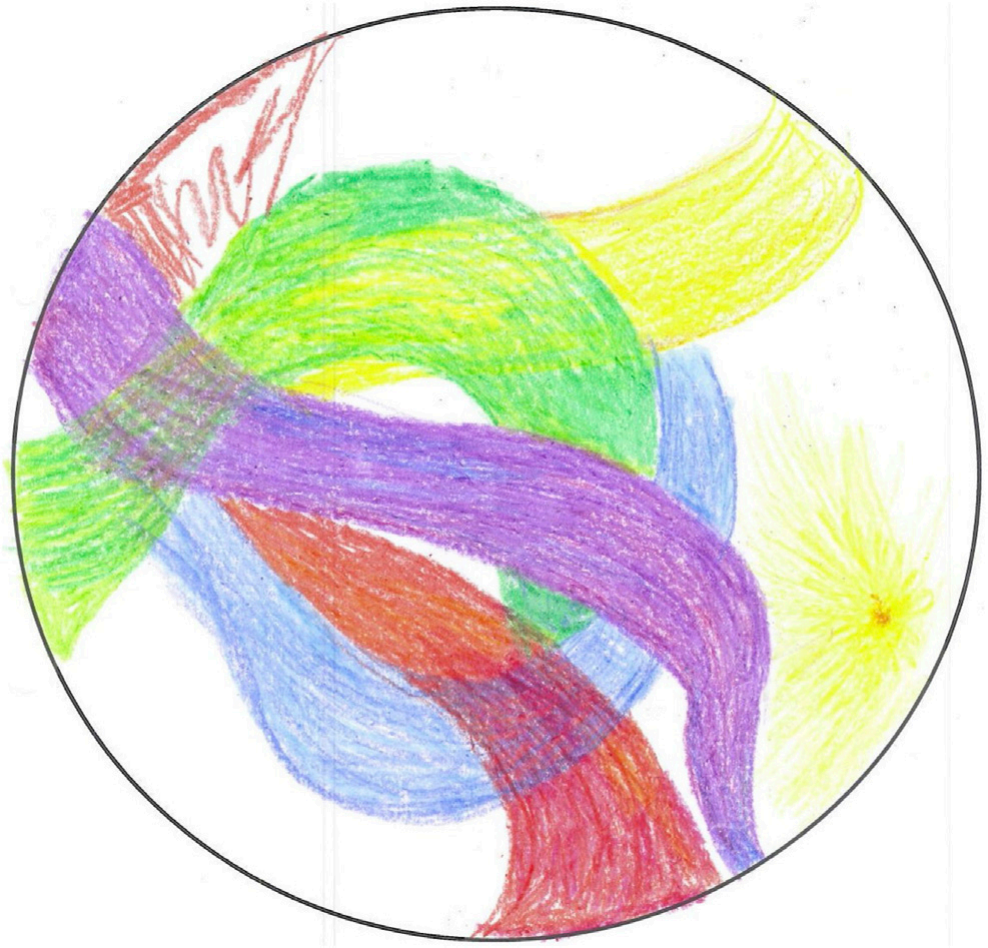
My inner vision became the universe, filled with colourful waterfalls, glistening like stars, not bound by gravity. They floated in the air and folded around each other. Totally quiet. The streams were infinite and beautiful. Report 15, female, CME, MEQ total 3.5.

*Report 15, female, CME, MEQ total 3.5*, see Figure 8

Some of the reports of beauty refer to participants experiences of reflecting on the nature of their consciousness and emotions.

*It gave me insights into consciousness as an abstract type of cake: Normally, unaffected, you see it as a beautiful object with all of its whipped cream and frosting, but throughout the experience it was as if you entered the different layers; all of them had their own contents to offer; and one only had to choose.*

*Report 13, male, CME, MEQ total 3.6*

*I think it is absurd that we try to examine this phenomenon. That we are scanning my brain to figure out what’s happening. I know that we can’t understand it, that we can’t put an equation on it and figure it out. I feel it is human arrogance, trying to tame all the beauty. I think, “Scan me then! We won’t get any closer to the truth!”*

*Report 31, male, CME, MEQ total 4.2*

Finally, many participants who had CMEs describe the beauty of the world and Universe around them, both natural and interpersonal. This is reflected in MEQ item 27 “sense of awe or awesomeness”

*With high spirits and a child-like excitement, I started to think about how the Norwegian adventurer Thor Heyerdahl crossed the Pacific Ocean on a raft to prove that life was big and beautiful. I see long realistic passages that made me laugh and feel a great inner joy.*

**FIGURE 9:**
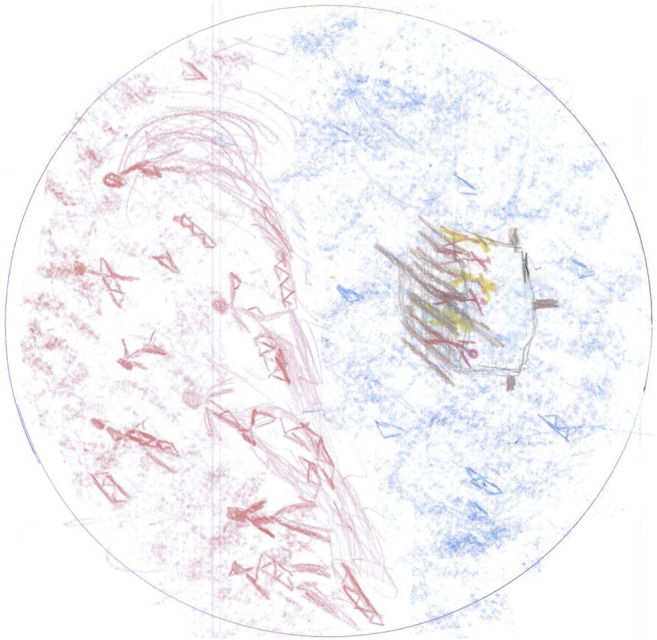
With high spirits and a child-like excitement, I started to think about how the Norwegian adventurer Thor Heyerdahl crossed the Pacific Ocean on a raft to prove that life was big and beautiful. I see long realistic passages that made me laugh and feel a great inner joy. Report 19, male, CME, MEQ total 3.8.

*Report 19, male, CME, MEQ total 3.8*, see Figure 9

The fifth highest tf-idf word was “simultaneous”. These quotes largely refer to a duality of experience (e.g., perceived disconnection of body and mind) wherein participants report feeling several, sometimes conflicting, emotions at the same time or reflect on their experience as it is ongoing. No specific items in the MEQ match these themes.

*In my thoughts, mentally, physically, and bodily. I desire the togetherness of being a pair, but simultaneously I feel a togetherness with the whole world and all people.*

*Report 12, male, CME, MEQ total 4.9*

*The next sequence was a mixture of thinking about different relationships and at the same time being present on a different level of consciousness, which transcended my body and my mind.*

*Report 16, male, CME, MEQ total 4.6*

*I open my eyes and cry with happiness. I am completely one with the past, the future and the present–stretched, but at the same time not in my body.*

*Report 31, male, CME, MEQ total 4.2*

#### Qualitative Reports of the Non-Complete Mystical Experience

Upon inspection, many of the words that had high tf-idf values from non-CME reports were driven primarily by one or two reports that used the words repeatedly. Thus, we did not feel that they provided a sound reflection of the themes within the non-CME. The only exception was the word “gloomy” which was used six times in non-CME reports, never in CME reports, and was reported by several individuals. In context this referred largely to interpretation of music.

*The physical insecurities I have about myself and the gloomy music made me feel uneasy*

*Report 7, female, non-CME, MEQ total 2.8*

## Discussion

### Summary

This study investigated how persistent positive effects of psilocybin attributed to the psychedelic experience are associated with the self-reported “mystical” nature of the experience in healthy volunteers. Analyses showed that the intensity and character of mystical-type experiences were associated with these, a relation which could be considered predictive as MEQ was collected approximately 3 months before PEQ. We provide qualitative lexical and pictorial descriptions of the psilocybin-induced Complete Mystical Experience (CME) describing themes such as connection to the Universe, familial love, beauty, and highlighting the importance of setting. Beyond replicating the relation between MEQ and PEQ in healthy volunteers, this paper shows that the character of mystical-type experience may be important for lasting positive effects in healthy volunteers and provides a qualitative description of the psilocybin experience in healthy volunteers that can be used to inform practice and future research around the administration of psilocybin in this population.

### Relation Between MEQ and PEQ

Previous research suggests that the mystical-type experience is qualitatively linked to patient outcomes (Belser et al., 2017; Swift et al., 2017; Nielson et al., 2018; Noorani et al., 2018) as well as being quantitatively linked to positive persisting effects in healthy volunteers (Griffiths et al., 2006; Griffiths et al., 2008; Griffiths et al., 2011; Griffiths et al., 2018; Schmid and Liechti, 2018) and patients (Garcia-Romeu et al., 2015; Griffiths et al., 2016; Ross et al., 2016; Davis et al., 2020). In this study we provide quantitative estimates of these effects in a population of carefully screened healthy Danish individuals. We also show that the degree to which these experiences were described as positively emotionally valanced, i.e., MEQ item 17 “Experience of ecstasy” or as feelings of connectedness, i.e., MEQ item 28 “Experience of unity with ultimate reality” were robustly associated with lasting positive effects. However, the degree to which the experiences were described as beyond typical comprehension of space and time, i.e., MEQ item 7 “Loss of your usual sense of space” or difficult to describe, i.e., MEQ item 10 “Feeling that you could not do justice to your experience by describing it in words” were not significantly associated with persisting positive effects. Importantly, the effect sizes for each subscale were similar, but the variance of the latter two subscales was too broad to conclude a significant association between these subscale scores and positive persisting effects. Although other papers have shown a relation between MEQ total score and persisting positive effects of psilocybin (Griffiths et al., 2011; Schmid and Liechti, 2018), this is the first paper to show a subscale-specific effect. Our results indicate that the subjective nature of the psychedelic experience is indeed linked to lasting positive outcomes, though it is argued that such links may be mere epiphenomena (Hibicke et al., 2020; Olson, 2021). Due to the lack of adequate controls and mechanistic antagonists, our study design is not suited to test whether the persisting positive effects of psilocybin are caused by the subjective or non-subjective effects of psilocybin. We did not observe a relation between peak plasma psilocin levels and MEQ score or persisting positive effects in the 20-participant subgroup for whom such data was available.

Given the above findings we may consider how to optimise the conditions that permit mystical-type experiences to unfold safely and allow patients to surrender to the experience (Aday et al., 2021), since this has been consistently associated with persisting positive outcomes. These may be modulated by parameters such as dose (Holze et al., 2021), choice of psychedelic compound, as preliminary literature suggests differential phenomenology between compounds (Richards, 1975; Shulgin and Shulgin, 1991; Shulgin and Shulgin, 1997; Metzner, 2015; Papaseit et al., 2018), or “set” and “setting” (Hartogsohn, 2017; Hartogsohn, 2021; Kettner et al., 2021; Perkins et al., 2021; Strickland et al., 2021). Notably, despite previous research showing that the scanning environment was strongly associated with negative reactions (Studerus et al., 2012), our mediation analysis showed no overall mediation effect of sub-project on persisting effects. A weak direct negative effect of sub-project 2 (comfortable environment) and sub-project 3 (MR scanner) was observed on lasting effects, compared to sub-project 1 (PET scanner). However, these associations do not survive multiple comparisons correction. Curiously, the term “MR” only appeared in CME reports and not in non-CME reports.

### Qualitative Findings

In this paper, we provide the first extensive qualitative descriptions of the subjective experience of healthy volunteers given medium-high doses of oral psilocybin in a modern research environment. The tf-idf analyses revealed themes in open-ended reports that aligned with items of the MEQ such as 1) connection with the Universe: “Experience of the fusion of your personal self into a larger whole.” (MEQ item 26), 2) A sense of infinity: “Feeling that you experienced eternity of infinity” (MEQ item 5) and 3) Wonderment/beauty: “Sense of reverence” (MEQ item 21) and “Sense of awe or awesomeness” (MEQ item 27) (Barrett et al., 2015). Despite playing prominent roles in the CME open-ended reports, certain themes emerged that are not described in the MEQ such as familial love, gratitude, and the simultaneous presence of contradictory feelings. The themes of family (Breeksema et al., 2020) and gratitude are also described in other qualitative reports (Watts et al., 2017; Noorani et al., 2018), and while not “mystical” in nature these may reliably co-emerge with the CME. None of the themes identified in the CME reports have connotations of the “challenging” or “dysphoric” experiences that have been reported in the qualitative literature of patient experiences (Belser et al., 2017; Swift et al., 2017; Nielson et al., 2018). Although this may be in part driven by the Positive Mood criteria of the CME, it still highlights the distinction between experiences in healthy volunteers and patients that deserves further investigation. We were not able to distinguish themes that surround non-mystical experiences, likely due to greater descriptive variability among these states relative to CME.

### Ineffability

One notable finding from the descriptive analyses was that even those who had non-CME had an average MEQ subscale Ineffability score of 3.9 out of 5, and every participant surpassed the threshold of 3 for CME relating to Ineffability. Some experts consider ineffability to be an essential component of an authentic CME (Papanicolaou, 2021), yet the idea that psychedelic phenomena are particularly ineffable is at odds with the idea that all phenomena are inherently ineffable, thus there is nothing special about psychedelia phenomena (Nagel, 1974). If we consider other phenomena to be “effable”, then describing psychedelic phenomena as ineffable can be considered scientifically pessimistic, and contribute to the culture of “psychedelic exceptionalism” (Johnson, 2021; Sanders and Zijlmans, 2021). What is it about a psychedelic experience, i.e., “Being in a realm with no space boundaries” (MEQ item 19) that is judged to be more ineffable than the inability to describe the colour blue, without resorting to comparison? It has been argued that “effability” is the product of shared experience and the subsequent production of language (Millikan, 2005), which is why “blueness” is easy to convey, at least to those who have experienced it (Jackson, 1982; Jackson, 1986). Therefore, the ineffability of these CME may reflect the novel nature of the experience to individuals without indicating any epistemic truth about the experience. If many people underwent high dose psychedelic experiences, then we might expect a new language to emerge that facilitates the description of such experiences between individuals. An example of this is the term “ego-dissolution” which is now widely used across psychedelic science (Lebedev et al., 2015; Nour et al., 2016; Preller and Vollenweider, 2018; Mason et al., 2020).

### Scientific Inquiry Into the “Mystical-Type Experience”

The focus of this paper is the investigation of the mystical-type experience, an area of inquiry with which some researchers have taken issue (Sanders and Zijlmans, 2021). Critics highlight the pervasive culture of mysticism in psychedelic science (including the MEQ itself) which may bias participants towards interpreting their experiences through a supernatural, unscientific, or spiritual lens where they may not have done so otherwise. It has been argued that the epistemic status of metaphysical conclusions drawn from psychedelic experiences are relevant to whether we should endorse psychedelics as a means to lasting positive psychological changes (Flanagan and Graham, 2017). Following psychedelic experiences, individuals may draw comfort from beliefs that are not veridical, which may be considered troublesome. This stance is known as the “Comforting Delusion Objection” and is discussed and objected to in detail by Letheby (Letheby, 2021). Nevertheless, psychedelic drugs reliably induce the sorts of experiences wherein individuals feel that statements such as “Certainty of encounter with ultimate reality” (MEQ item 9) and “Sense of being at a spiritual height” (MEQ item 15) accurately describe a component of their experience (Griffiths et al., 2006; MacLean et al., 2012; Barrett et al., 2015). As participants feel justified in describing their experiences with such language, the framework of “mystical-type experiences” is a useful one for describing a cluster of co-occurring experiences, independent of ontological judgement of their content, especially given the apparent predictive utility of such experiences.

### Limitations

In the latent variable model analyses, we attempted to control the type-1 error at its nominal level using a method described by Ozenne, Fisher, and Budtz‐Jørgensen (Ozenne et al., 2020). Additionally, we accounted for individuals who took part in two projects using robust standard error (section 6 of Ozenne, Fisher and Budtz-Jørgensen). Despite these corrections, it is still noteworthy that the type-1 error may be inflated by the sample size and we urge independent replication with larger samples, especially given these scales are widely used in psychedelic research.

This study had a low sample size of 28 participants and 35 observations, was not blinded, and did not account for expectancy effects which have been theorised to mediate some of the persisting positive effects of psychedelics (Muthukumaraswamy et al., 2021). Rigorous future work should aim to replicate these findings with such controls and evaluate the role of expectancy in the intensity and character of mystical-type experiences induced by psilocybin. By performing a mediation analysis, it may be possible to delineate the degree to which expectancy is either related to persisting positive effects and whether independent of or via MEQ. The PEQ specifically asks participants to evaluate changes that are *due to* the experience, thus further work could aim to investigate the subjectively experienced causal link between the content of the experience and persisting positive effects. Additionally, we do not collect data on whether participants recreationally consume psilocybin between administration within the study and 3-month follow up, which has the potential to bias evaluation of persisting effects.

The PEQ scores that we report are similar in value to those reported by a group based in Basel, Switzerland for a comparable dose of LSD (Schmid and Liechti, 2018). Curiously, these scores are equal to the PEQ scores reported for the placebo group from studies at Johns Hopkins in the United States, and far lower than the PEQ scores reported following similar doses of psilocybin (Griffiths et al., 2011). This suggests that there may be a relevant difference between studies, such as recruitment basis, age, preparation, setting, and integration practices, or cultural differences that should be taken into account when considering the generalisability of these findings. Notably, the PEQ has not been robustly compared to other measures of persisting psychological change, or undergone factor analyses. Our sample is drawn from three separate settings, namely PET scanning, a comfortable environment, and MRI scanning. This adds heterogeneity to our sample, though our mediation analysis did not show a significant effect of setting on PEQ either directly or via MEQ. Nevertheless, analyses comparing the mystical-type experience to persisting positive effects could be repeated with a more homogenous setting.

Due to the lack of generalisability of the terms within the corpus of non-CME reports, we did not feel that these were sufficient to provide a qualitative characterisation of non-CME. The only exception was “gloomy” that referred in several cases to the music, a sentiment that has been identified in previous qualitative psychedelic research (Watts et al., 2017).

Qualitative analyses are often limited by biased selection of quotes that fit the narrative of the paper (Galdas, 2017). We attempted to control for this by utilising a natural language processing procedure, tf-idf, to delineate themes without a priori criteria. However, quotes selected after this filtering step are not entirely unbiased, as not all quotes are reported. Additionally, although tf-idf analyses account for total corpus length, they are susceptible to bias from a single report containing a specific word repeatedly. We attempted to control for this bias by manually searching to ensure each word described was used in multiple reports. Tf-idf analyses are limited in cases where there are only two document bodies as a single incidence of the term in both bodies results in a tf-idf value of zero. Therefore, the high tf-idf scores reported above represent the most frequently used terms that only appear in the CME reports, though this may also be considered a strength of these findings. Further collection of these open-form reports on the night of the session will allow for more nuanced natural language processing of the data, as there were several analyses that we were unable to perform due to a small sample (e.g., thematic analyses, n-gram analyses, splitting into smaller subgroups). These may provide a deeper understanding of the phenomenology of the psychedelic experience.

### Conclusion

In this paper we show a relation between the intensity of the psilocybin-induced mystical-type experience and lasting positive psychological effects and extend previous work by showing that the MEQ subscales Mysticality and Positive Mood were more closely associated with lasting positive effects than Transcendence of Time and Space or Ineffability. This suggests that the phenomenology of the psychedelic experience is relevant to lasting positive effects. Additionally, we provide the first extensive qualitative descriptions of orally administered psilocybin in healthy volunteers revealing themes including universal connectedness, experience of beauty, and familial love that may be used to inform future research utilising psilocybin in healthy volunteers.

## Data Availability

Data may be made available on request to the CIMBI database. Requests to access the datasets should be directed to https://www.cimbi.dk/.
