# Supplementary material for: Psilocybin-Induced Mystical-Type Experiences are Related to Persisting Positive Effects: A Quantitative and Qualitative Report
Source: Front Pharmacol. 2022 Mar 9;13:841648. doi: 10.3389/fphar.2022.841648 (PMC8959755; doi:10.3389/fphar.2022.841648)
Supplement: Supplementary file 2 [file DataSheet1.docx]

**Psilocybin-Induced Mystical-Type Experiences are Related to Persisting Positive Effects: A Quantitative and Qualitative Report**

Supplementary Material

# Supplementary Data

## Supplementary Tables

**Supplementary Table 1** Individual MEQ and PEQ subscale scores

| **Subscale Mean (SD)** | **All** | **CME** | **Non-CME** |
| --- | --- | --- | --- |
| MEQ total | 3.6 (0.9) | 4.2 (0.5) | 2.7 (0.6) |
| Mysticality | 3.3 (1.2) | 4.0 (0.6) | 2.1 (0.9) |
| Positive Mood | 3.6 (0.9) | 4.1 (0.7) | 2.8 (0.8) |
| Transcendence of Time and Space | 3.9 (1.0) | 4.5 (0.5) | 3.2 (0.9) |
| Ineffability | 4.3 (0.7) | 4.5 (0.7) | 3.9 (0.5) |

**Supplementary Table 2** MEQ total and subscale scores as split by CME and non-CME

**Supplementary Table 3** (LVM mediation analysis results) and **Supplementary Table 4** (Complete descriptions of LVMs) are provided as standalone excel files

## Supplementary Figures


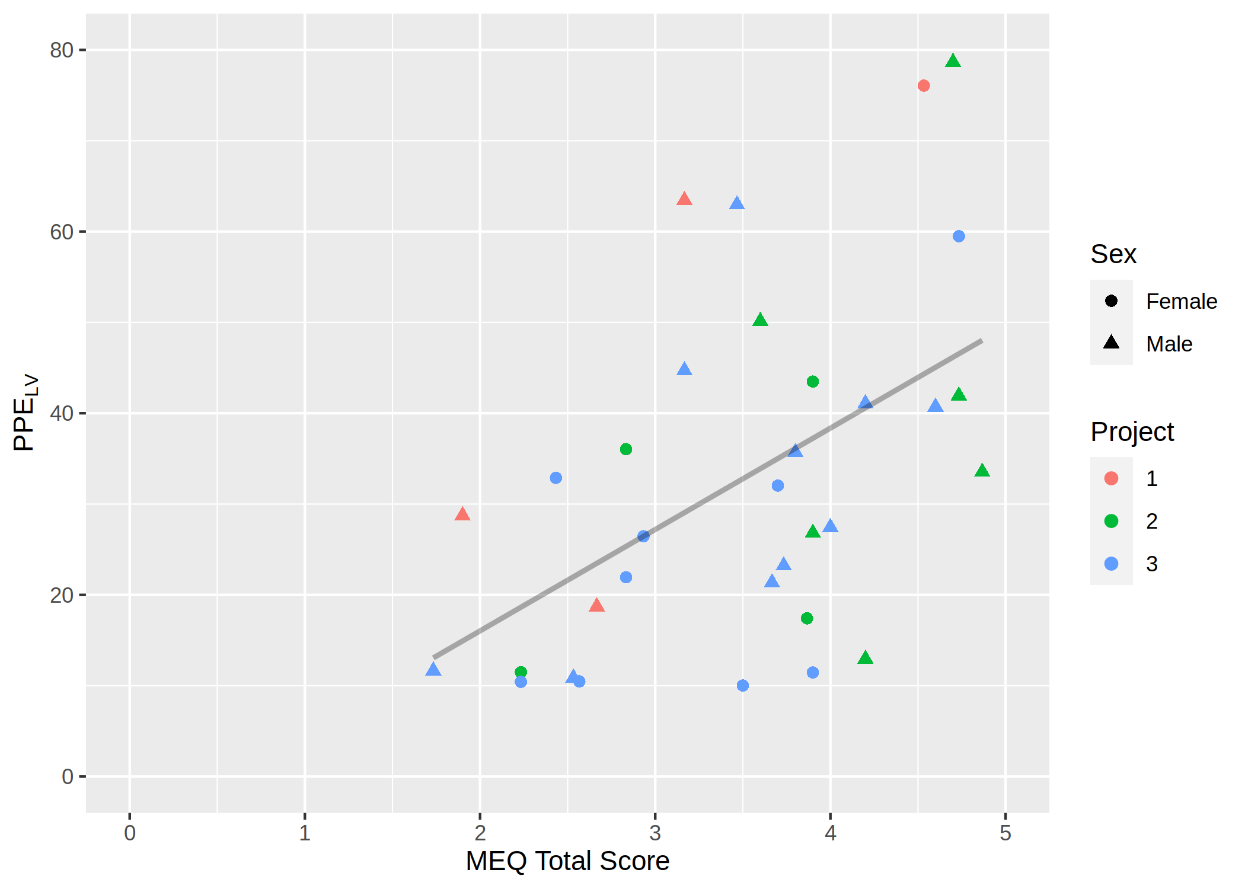


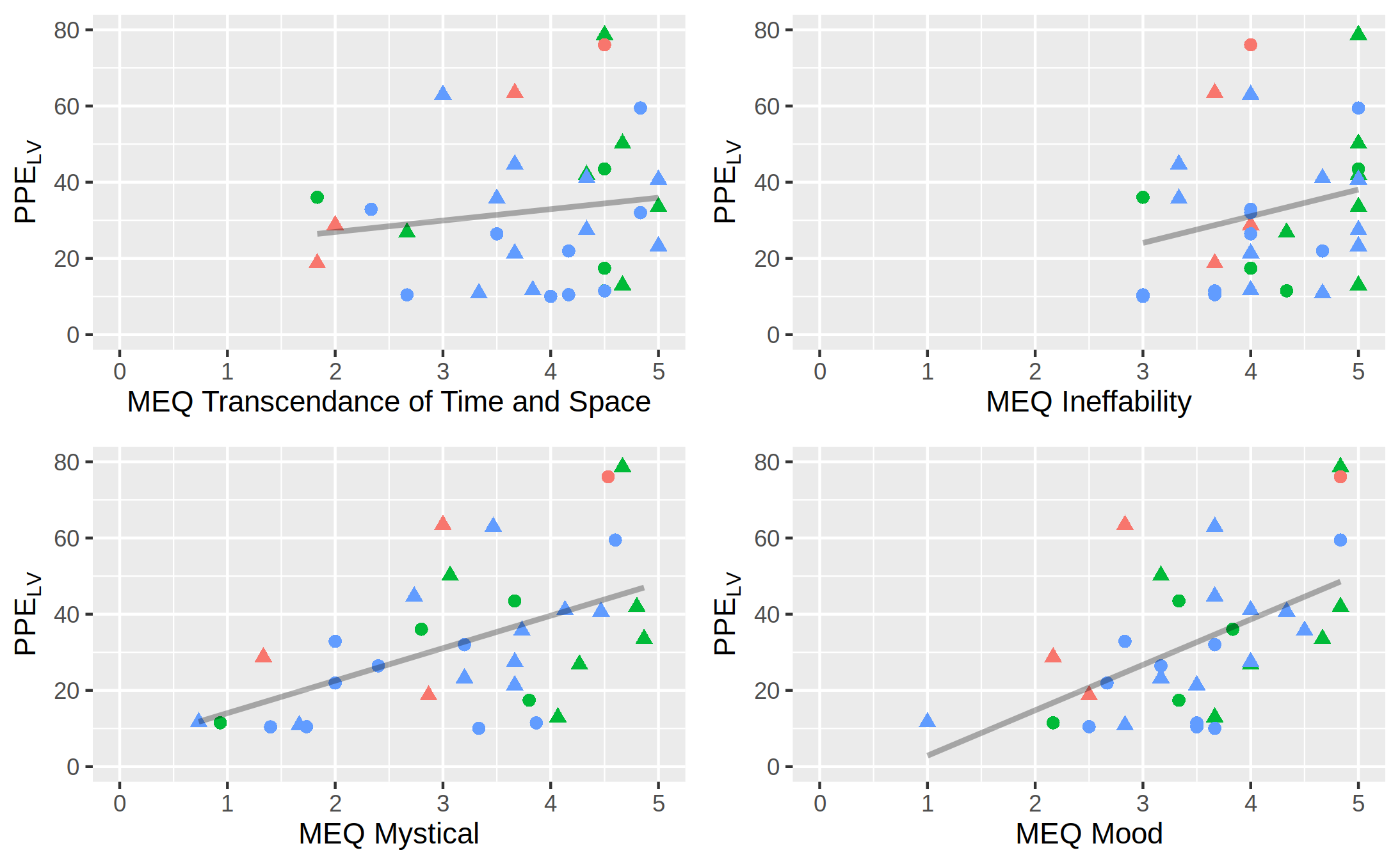
 **Supplementary Figure 1** Scatter plots showing MEQ total score or subscale scores and PPE_LV_ and a univariate linear model. Colour denotes subproject involvement and shape denotes the sex of the participant. No formal statistics were evaluated as these plots are only for visualisation purposes.


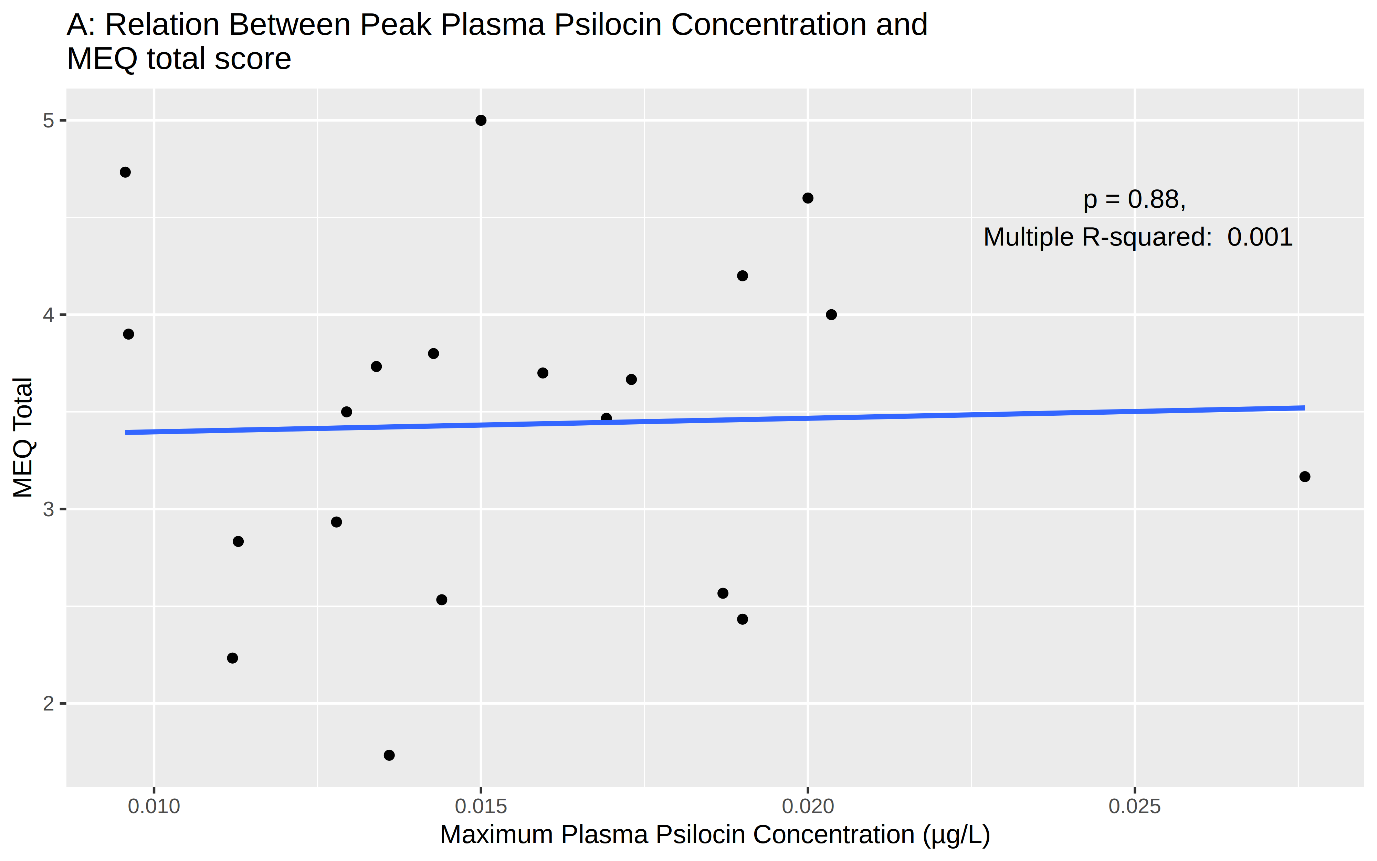


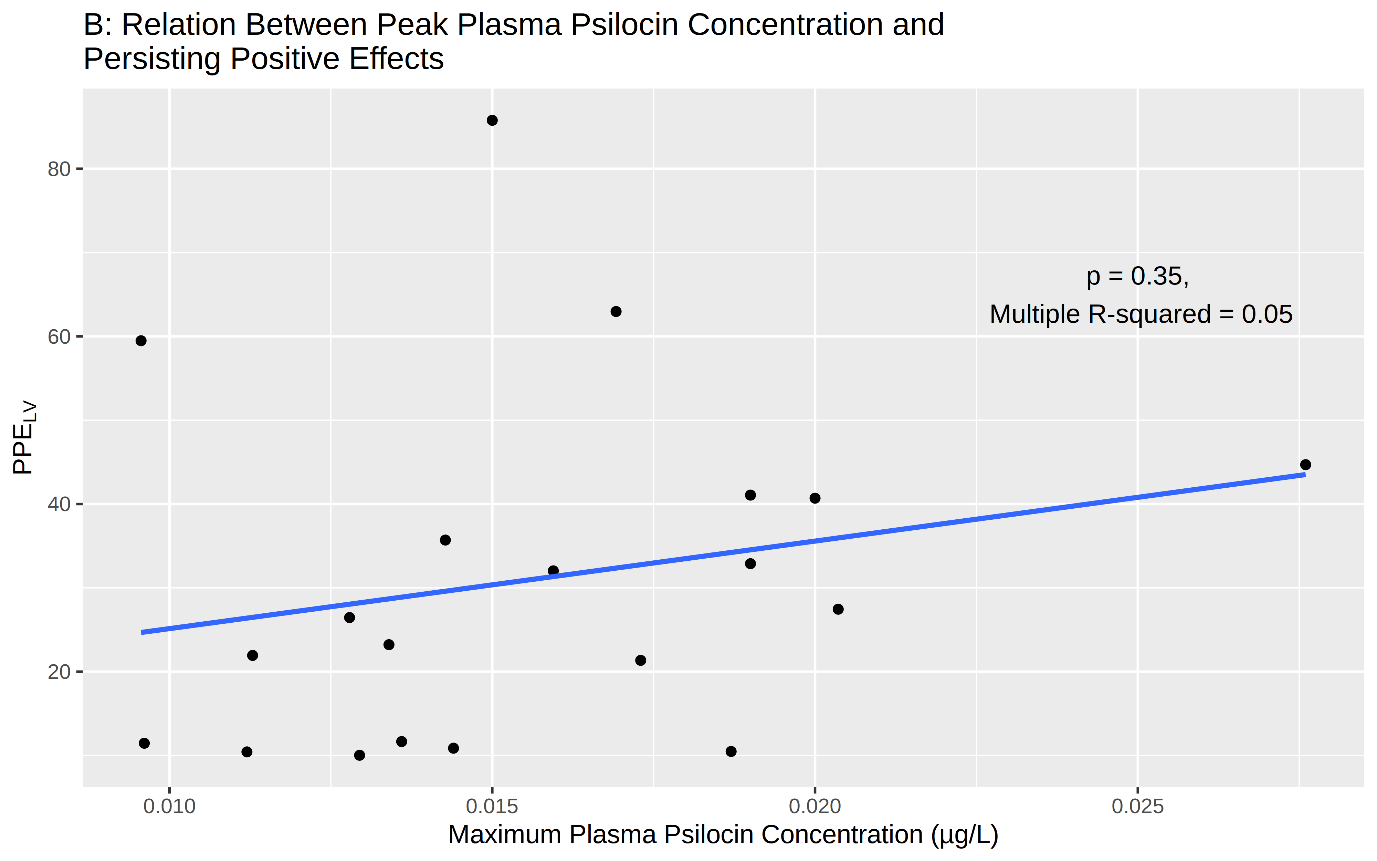


**Supplementary Figure 2** Scatter plots showing (A) MEQ total score or (B) PPE_LV_ and Maximum plasma psilocin concentrations for the 20 participants from project 3 with complete data. Neither association is statistically significant.
